# Pattern of Corrosive Ingestion in Southwestern Saudi Arabia

**DOI:** 10.4103/1319-3767.41744

**Published:** 2009-01

**Authors:** Ali M. Al-Binali, Mohammmed A. Al-Shehri, Ismail Abdelmoneim, Ali S. Shomrani, Suliman H. Al-Fifi

**Affiliations:** Department of Pediatric, College of Medicine, King Khalid University, Abha, Kingdom of Saudi Arabia; 1 Department of Family and Community Medicine, College of Medicine, King Khalid University, Abha, Kingdom of Saudi Arabia; 2 Pediatric Department, Aseer Central Hospital, Abha, Kingdom of Saudi Arabia

**Keywords:** Children, corrosive, ingestion

## Abstract

**Background/Aims:**

Ingested corrosive material is a major pediatric emergency all over the world. The corrosive material can cause damage to the digestive tract, ranging from minor injury to strictures, and sometimes even death. We aimed to review the pattern of corrosive ingestion in children who had been admitted to Aseer Central Hospital in the Southwestern region of Saudi Arabia.

**Methods:**

This is a retrospective study of all children who had been admitted with a history of corrosive ingestion to Aseer Central Hospital over a period of five years period from 1990 to 1995. The records of 72 patients (38 males and 34 females) were reviewed. The data included age, sex, time lapse till admission, action taken by parents, presenting symptoms, general management given to the child, barium study, endoscopy, and the postcorrosive ingestion outcome of the child.

**Results:**

The mean age of the pediatric patients was 28 ± 20 months. Different types of corrosives were encountered. The most common type was 5.25% hypochlorite in 36 patients (50%), kerosene in 12 patients (16.7%), caustic soda in nine patients (12.5%), hydrogen chloride and N-alkyl dimethyl benzyl ammonium chloride (HC and ADB) in eight patients (11.1%), and other material in seven patients (9.7%). Endoscopy was done in 30 patients (31.7%), 14 of whom were abnormal. Barium swallow was performed in 11 patients; five of them showed strictures that required frequent dilatation whereas one needed interposition surgery.

**Conclusion:**

Corrosive injury is still a major pediatric emergency among young children. It carries a major risk of complications (mainly stricture) and requires standardized management based on evidence-based medicine.

Caustic injury to the digestive tract remains a significant medical and social concern despite various efforts to minimize its hazards.[1] It is potentially capable of burning the esophagus and the stomach. Young toddlers who tend to investigate their surroundings and are unaware of many dangers, are especially prone to these kinds of accidents.[2,3]

Agents of pH < 2 or > 12 are extremely corrosive. The damage to the gastrointestinal tract ranges from mild to extensive injuries.[1]

A decline in the incidence of caustic injuries has been noted with an estimated incidence of 5,000–15,000 cases per year in the USA.[4] However, an increase in caustic injuries has been reported in other countries such as Turkey.[4] There is scanty information about corrosive ingestion in Saudi children.[5–7]

The aim of this review is to study the pattern of corrosive ingestion in children admitted to Aseer Central Hospital in the southwestern region of Saudi Arabia so as to have a baseline for future comparison.

## PATIENTS AND METHODS

The Aseer region (population = 12,00,000), located in the southwest of Saudi Arabia, covers an area of more than 80,000 km². Aseer Central Hospital is a five-hundred bed hospital with an annual average of 20 000 inpatients and 130, 000 outpatients.

This is a retrospective study of all patients admitted to Aseer Central Hospital over a five year period from 1990 to 1995. The admission and discharge logbook was reviewed and any patient admitted with corrosive ingestion was included in the study.

The records of 72 patients (38 males and 34 females) were reviewed. The data included age, sex, time lapse till admission, action taken by parents, presenting symptoms, general management given to the child, barium study, endoscopy, and the postcorrosive ingestion outcome of the child. The endoscopy findings were graded according to the modified classification for describing caustic injury to the esophageal lumen[8] [Table 1].

**Table 1:** Classification of endoscopic grading of esophageal injuries

Normal	No endoscopic damage
First degree	Mucosal erythema
Second degree	Erythema with noncircumferential exudate
Third degree	Circumferential exudates
Fourth degree	Circumferential exudates with esophageal wall perforation

The information pertaining to individual cases was collected in a standardized data sheet. The accuracy of the tabulated data was cross-checked by a second individual to maintain consistency and accuracy. The findings of the Barium swallow study, chest X-ray, and endoscopy findings were recorded based on the chart reports.

## RESULTS

**Figure 1:**
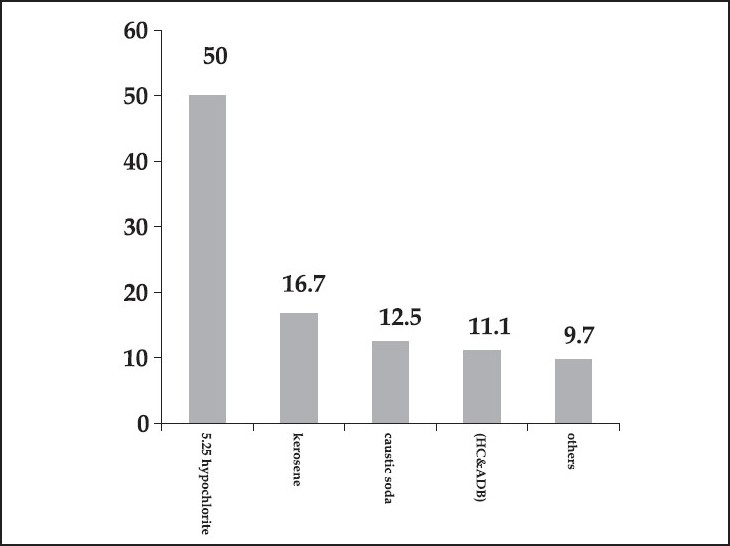
Frequency of corrosive ingestion among studied children

A total of 72 files were reviewed: 38 males (53.5%) and 34 females (46.5%); the mean age was 28 ± 20 months. Different types of corrosives were encountered during the review. The most common type was 5.25 hypochlorite in 36 patients (50%), followed by kerosene in 12 patients (16.7%), caustic soda in nine patients (12.5%), and HC and ADB in eight patients (11.1%). Other materials were encountered in seven patients (9.7%) including fertilizer (1), thinner (1), sodium hydroxide (1), insecticide (1) and three patients had ingested nonspecified corrosives [Figure 1].

No action was taken by the parents of 65 patients (90.2%). Vomiting was induced in four patients (5.6%) and milk was given to three patients (4.2%).

The documented clinical presentations were vomiting in 32 patients (44.4%), breathing difficulties in 12 patients (16.7%), oral ulcers in 12 patients (16.7%), fever and cough in nine patients (12.5%), dysphagia in three patients (4.2%), salivation in six patients (8.3%), and chest pain in one patient (1.4%).

General management included IV fluid administration and nothing per OS in most of the patients admitted to the hospital, however, there was no standardized care regarding the use of antibiotics, hydrocortisone, H_2_-receptor antagonists (H2 blocker) blockers, or protone pump inhibitors. Most of them (77.7%) received some form of antibiotics. Only seven patients (9.7%) received H2 blockers but none received protone pump inhibitors. Forty-nine patients (68.1%) received IV hydrocortisone, the duration and the dose of which were not according to specific standards or guidelines.

Endoscopy was done in 30 patients (31.7%) with variable time lapses from admission (mean 37 ± 28 hours). Sixteen of these patients (53%) were reported to be normal, although the endoscopy was abnormal in 14 out of these 16 patients (47%). The endoscopic abnormality ranged from grade I injury in nine patients (64.2%), grade II in two patients (14.2%), and grade III in three patients (21.4%).

Chest X-ray was taken in 51 patients (70.8%) and was reported to be abnormal in 12 of them (31.3%). Barium swallow was done in 11 patients (15.2%) to confirm strictures. The study showed strictures in five patients (two patients after drain opener ingestion, two patients after caustic soda, and one patient after HC and ADB ingestion). The five patients who showed strictures in their Barium study, required a different modality of treatment ranging from frequent dilatation to interposition surgery in one patient. The mean follow-up period was 3.7 ± 9.1 months. No death was reported in this study.

## DISCUSSION

Injury to the gastrointestinal tract, secondary to corrosive ingestion, is a worldwide pediatric emergency problem.[8] Children younger than five years of age constitute the highest risk group for accidental corrosive ingestion, with the peak age of risk being two years.[9,10] Children at this age have well-developed skills to locate and drink liquids, but are unable to discriminate edible liquids from toxic ones.[11]

In our study, 72 children were admitted to the hospital over a period of five years. The male to female ratio was 1.1:1 with a mean age of 2.3 years. The most encountered corrosive ingestion was 5.25% hypochlorite which constituted 50% of the accidents. This finding is different from those of other reports where alkalis were the most frequently encountered corrosives.[11–13] Alkali ingestion in our group ranged from pipe drainer, which comprised 25% of all cases, compared to 71% reported by Huang *et al*.[11] and 100% by Contini.[14]

Endoscopy was done in 30 patients (31.7%) which is different from other reports in which all[11,12] or 84%[15] of children had been scoped within 48 h. In our study, among those patients in whom gastroscopy was done, 14 of them (47%) were found to be abnormal. The abnormality ranged from grade I in nine patients (64.2%), grade II in two patients (14.2%), and grade III in three patients (21.4%), compared to 20% with normal results, 60% grade I– II, and 40% grade IIb–III observed by Yu *et al*.[12] Other reports showed 61% grades I and II, grade III (19%), and grade IV (17%).[1]

Strictures were evident after barium swallow in five patients (6.9%), although the study was done only for 11 patients (15.2%). Five of those patients responded to frequent dilatation except one who required colonic interposition surgery. Our finding is different from other reports,[15] in which 72.6% developed stenosis initially, and 15.8% of them healed with no effect on the esophageal diameter. Others reported development of strictures in15 out of 24 patients (63%).[11] On the other hand, no stricture was reported in the pediatric age group by others.[1] We did not observe any deaths among our patients as compared to 0.8% by others.[15]

In this study, we found that corrosive injury is still a major pediatric emergency among young children and it carries a major risk of complications (mainly stricture). The efforts and coordination of pediatricians are needed to educate public sectors about the great risks of such substances and the best way in dealing with them, especially after their ingestion. Another important point is to unify an acceptable management approach for such patients based on the clinical evidence.
